# Syncope—Asphyxia—Poisoning

**Published:** 1865-10

**Authors:** 


### SYNCOPE—ASPHYXIA—POISONING.

In the *Dublin Med. Press* Dr. Brown Sequard publishes a lecture on the brain and nervous system, from which we condense some of the closing remarks, eminently interesting and practical:

In cases of syncope—not death, but those conditions which are on the verge of death, and which lead to death, if nothing is done to relieve them—there are very frequently means of restoring life. In animals, very frequently, though the heart is quite stopped, we can by simply pressing on the sternum, and by giving a hard push to the heart, make it beat. It will not beat long if the cause of the syncope is a powerful one; but beat it will, and if the cause of irritation is continued, it will continue to beat, and in that way the patient may be often revived. This is not all. If there is added another most powerful cause of revival, and which is directly the reverse of what John Hunter did upon himself, when he found he was in a state of syncope one day at College—if instead of breathing as quickly as possible, the patient's breathing is stopped altogether, just as if we were trying to kill him by suffocation, we revive him by producing a state of asphyxia, the patient is saved, he will have a struggle, and will come out of it very quickly. Nothing is more powerful to make the heart beat than an accumulation of carbonic acid in the blood. There are other features about syncope of great importance. If there is little blood circulating, you may in a moment throw something like one or even two pounds of blood into the heart by simply pressing on the four main arteries of the body. If you press those four arteries you prevent circulation going on in them, and at once an immense quantity of blood returns from the venous system to the trunk, and there is an immediate revival.

In regard to *asphyxia*, experiments show clearly that of two animals, one of them with its temperature, both dipped into water at the same time, the one having its temperature very low will survive the other twice, three times, and sometimes even five times; the duration of life under water being extended sometimes to twelve or fifteen minutes. *The greater the previous diminution of temperature the longer the duration of life.* There is another fact which is a very interesting one. It is well known that persons who have fallen into very cold water have in many cases been drawn out and revived after a number of minutes' immersion. Now, in experiments performed upon animals by applying galvanism to the part, so as to stop the heart's action, which is just the effect a fall into water will produce, we find life will last much longer, the animal will be able to survive a much longer stoppage of the heart's action from having had an attack of syncope just before the asphyxia. This case, then, is exactly the reverse of the former. In one, syncope was cured by asphyxia, in the other asphyxia is less mortal, because syncope previously existed.

In cases of death by asphyxia, if the temperature is low, there is one fact very similar to what we see in cases of sudden emotion producing arrest of the heart's action when the individual falls into cold water, and that is, that in the two cases—diminution of temperature, or dipping into water—the heart beats very much slower. Any patient attacked with asphyxia, whose temperature is much diminished, has a slow beating of the heart, and whose blood is red, is not exactly an asphyxiated patient; there is a mixture of syncope and asphyxia, and the patient has much more chance to recover. If you try to raise the temperature of such a patient, you run the risk of killing him.

Poisoning is often the cause of death by producing such diminution of temperature as is incompatible with life. Take, for instance, two animals which have been poisoned with the same quantity of opium. Supposing the temperature to be cold in the room, lay them both on a table, one covered carefully with warm clothes, the other exposed to the cold, you find, *cæteris paribus*, that the one which is kept warm will survive, while the other will die. This fact we find with almost every poison of an organic nature, that there is considerable diminution of temperature produced, if hot *per se* sufficient to cause death, enough, at any rate, to add a powerful cause to the other causes existing. This diminution of temperature is a feature which we can fight against, and it is therefore of the utmost importance in cases of poisoning to use every means to keep up the temperature of the body.

